# Outcome of Vaginal Progesterone as a Tocolytic Agent: Randomized Clinical Trial

**DOI:** 10.5402/2012/607906

**Published:** 2012-05-23

**Authors:** Soraya Saleh Gargari, Malihe Habibolahi, Zahra Zonobi, Zahra Khani, Fatemeh Sadat Sarfjoo, Atefeh Kazemi Robati, Roja Etemad, Zohreh Karimi

**Affiliations:** ^1^Feto-Maternal Unit, Mahdiyeh Hospital, No. 16, Fadaieaneslam Street, Shoush Avenue, Tehran 1185817311, Iran; ^2^Department of Obstetrics and Gynecology, Mahdiyeh Hospital, Shahid Beheshti University of Medical Sciences, Tehran 1185817307, Iran

## Abstract

Vaginal progesterone has a potential beneficial effect in postponing of preterm labor by suppression of prostaglandins cascades. Although different studies evaluated the use of progesterone for preterm birth, the exact effect of which on prolongation of pregnancy remains unclear. Seventy two women who underwent preterm labor were managed by magnesium sulfate. Then they were randomly assigned to continue pregnancy either by applying vaginal progesterone (400 mg) until delivery or without using any drug. Gestational age mean at the time of delivery (*P* = 0.039) and postponing delivery mean time (*P* = 0.048) were significantly higher in progesterone group. Comparison of neonatal outcomes between two groups of patients showed meaningful benefits of progesterone in increasing of neonatal weight, reduction of low birth weight babies, and lowing neonate admitted in NICU.

## 1. Introduction

The frequency of preterm delivery contributes a relatively small proportion of total births (5–11%) [1, 2], while it is associated with excess of 70% of the total perinatal mortality in developed countries, when excluding deaths related to congenital anomalies [3, 4]. 

Moreover, preterm delivery prevention is well recognized as a major strategy for immediate and long-term costs reduction after discharge from the hospital and childhood morbidity [5–7].

Because of multifactorial (social, behavioral, and biological) causes in preterm delivery, efforts in prevention measures have not been successful so far [8]. Furthermore, occurrence of spontaneous preterm birth increases 12–22% in recent reports [9, 10].

Therefore, various tocolytic agents have been used to postpone preterm uterine contractions for at least 48 hours which allow maximal effect of antenatal steroid administration to assist in fetal lung maturation and maternal transportation to a center with neonatal intensive care unit (NICU) [11].

From early 1960s, progesterone as a tocolytic agent was used for the preventing of preterm birth [12]; however, relationship between progesterone withdrawal and the onset of labor is a considerable debate [13, 14].

Progesterone can affect preterm labor reducing contractility and inflammatory processes [15–19].

Although previous studies reported that serum level of progesterone following its vaginal administration is lower comparing to intramuscular administration, optimal route of progesterone administration in women undergone preterm labour is yet being considered. Moreover, there are controversial documents about the benefit of vaginal progesterone on spontaneous preterm birth [20]. We designed randomized clinical trial study for clarification of the effect of vaginal progesterone on spontaneous preterm birth. 

## 2. Methods

### 2.1. Patients

After approving trial protocol of study in Ethics and Research, Committee of Beheshti University of Medical Sciences and registration of clinical trial in Iranian registry of clinical trial (IRCT: Code no. 7291), composed of specialized antenatal clinics caring for preterm labour, women were enrolled in the study for a period of 3 years from 2007 to 2010.

An RCT study was carried out during a 3-year period, from March 2007 through March 2010 on all singleton pregnancies at ≥24 and <34 weeks complicated with preterm labor attending labor ward in Mahdieh Tertiary Care Hospital affiliated to Shahid Beheshti University of Medical Sciences in Tehran, Iran. The aim was to assess the efficacy of maintenance vaginal suppository of cyclogest therapy in patients with cervical length less than 15 mm by vaginal ultrasound that acute preterm labor was successfully controlled by intravenous magnesium sulfate. The criteria used for the diagnosis of acute preterm labor included persistent uterine contractions (e.g., at least four every 20 minutes or eight every 60 minutes), cervical dilation of 1 to 3 cm, effacement exceeding 50 percent, a change in cervical dilation, or effacement detected by serial examinations [21, 22]. Gestational age was assigned based on the last menstrual period (LMP) if it was confirmed by ultrasound or ultrasound alone when LMP was unknown. Intrauterine growth restriction (IUGR) was defined as birth weight less than the fifth percentile [23].

Exclusion criteria were preterm premature rupture of membranes, premature termination for obstetric indications and fetal anomaly, vaginal bleeding, polyhydramnios, fetal anomalies, suspected chorioamnionitis or intrauterine growth retardation (IUGR), and concomitant cardiovascular disease of woman (e.g., preeclampsia, gestational or chronic hypertension).

### 2.2. Procedures and Outcomes

All these patients after admission to the labor ward initially received intravenous magnesium sulfate, a 6 gr loading dose followed by maintenance dose of 2 gr/hour, to stop uterine contractions. All patients were given betamethasone (12 mg) and repeated after 24 hours and intravenous ampicillin (2 gr) every 6 hour until the results of group B streptococci vaginal cultures were received.

The patients were weaned from intravenous magnesium sulfate after uterine contractions were stopped for 24 hours. All eligible participants gave written informed consent before study entrance. The patients were assigned for maintenance vaginal suppository of cyclogest 400 mg every night or without using any drug randomly which was continued until labour or 37 weeks of gestation. Permuted blocks were scheduled to be given a participant number randomly corresponding to a specific treatment (either active or no drug packs). During treatment procedure, all study personnel and participants were blinded except statistician who did not have any contact with study participants.

Weekly vaginal sonography to assessment of cervical length was performed for all patients. Until time of delivery, women were followed up for primary outcomes which were recorded such as duration of pregnancy, gestational age of delivery, type of delivery, duration of stay in hospital, and intrauterine fetal death. Moreover, neonatal death, neonatal unit admission, and duration of neonatal unit care were compared between two groups.

### 2.3. Statistical Analysis

Statistical analysis on collected data was processed by SPSS software (Version 16.0). Wilcoxon signed paired test was used for before-after analysis of these dependent samples, and differences were considered statistically significant when the *P* value was < 0.05. Student *t*-test and occasionally the Mann-Whitney test were used for comparison of continuous variables. Linear regression was recruited if the association of two continuous variables was analyzed. Calculation of *P* < 0.05 and odds ratios (ORs) with 95% confidence intervals (CIs) were estimated as a significant difference.

## 3. Results

Because of withdrawal of consent or being not traceable after moving out of study area, 38 women were lost to followup in both groups.

Therefore, primary outcome of 72 patients and controls was available for analysis. There was no significant difference in demographics and clinical characteristics of the groups (Table 1). Mean gestational age at the time of delivery (36.2 ± 1.4 versus 34.1 ± 1.5; *P* value = 0.039) and mean time of postponing delivery (4.0 ± 1.5 versus 1.4 ± 0.2; *P* value = 0.048) were significantly higher in patients who used vaginal progesterone (Table 2). However, the mean of cervical length and dilatation were not changed comparing groups after treatment. Necessity to Cesarean section was more probable in control group (19 patients; 26.5%) rather than cases (16 patients; 22.2%), but this difference was not meaningful. There was no record for adverse events which need medical care comparing two groups.

Table 3 shows the neonatal outcomes comparison between two groups of patients. None of the women was reported with an intrauterine death in both groups. Neonatal mean weight was 2950.6 ± 420.3 in progesterone group and 2628.0 ± 385.1 in no-drug group (*P* value < 0.001). Only eight babies fulfilled criteria of low birth weight, in contrast to 26 born in no-drug group (*P* < 0.001). The number of neonates admitted in NICU had significant difference between groups (8.3% versus 23.6%; *P* value < 0.001). However, the *P* value for the formal test of correlation between the type of treatment and neonatal death was not significant (*P* = 0.082).

## 4. Discussion

Preterm birth remains a significant cause of early neonatal mortality and specific morbidity associated with prematurity [17, 24].

Based on this study finding, vaginal progesterone may increase the demur time of delivery if such efficacy confirmed by further investigation vaginal progesterone can eliminate risk of hospitalization in the first year of life, learning difficulties [25, 26], behavioral problems [27, 28], and burden of economic consequences [5, 29].

There is this hypothesis, “suppression of uterine contractile activity [15, 16, 30] through inhibition of the calcium-calmodulin-myosin light chain kinase system [16, 30, 31] is the main role of progesterone in continuation of pregnancy [31–33].”

Moreover, negative effect of progesterone on prostaglandin production and interaction at the fetoplacental unit may be a probable mechanism for clogging preterm birth [15, 18, 19].

Although the results of this study were the result of 400 mg vaginal progesterone gel use, with ethical limitation, we afford routine tocolytic therapy to both case and no-drug groups. Moreover, the route of progesterone administration may affect pharmacokinetics and peak blood concentrations time (3 to 8 hours for 100 mg vaginal progesterone). The potential lack of delayed local absorption and marginal blood peak level can be proved by high-concentration use of vaginal gel. However, there were similar effects of both intramuscular and vaginal progesterone in several systematic reviews of randomized controlled trials. Similarly to date, the optimal dose of vaginal preparations (ranging from 90 to 400 mg daily) [20, 34] and optimal time to commence therapy (24 to 28 weeks of gestation) [20, 35, 36] vary considerably across studies. Obviously, selection of proper dosage and timing for this study was resultant of all previous lucrative studies.

There is more limited information available relating to definitive health outcomes of vaginal progesterone in women presenting with symptoms or signs of threatened preterm labour; therefore, an ongoing trial (by Matrinez et al.) assessing this role will contribute information in the future as well as our studied parameters. 

Although we observed significant advantages of vaginal progesterone for the remainder of pregnancy on gestational age at the time of delivery and time of postponing delivery of women with spontaneous preterm labour, this finding may not be repeated in women with other risk factors like short cervix, past history of spontaneous preterm birth, and multiple pregnancies [37–39].

During followups, there was no report received from women complaining from sideeffects of vaginal progesterone (headache, nausea, breast tenderness, and coughing) [40] in consistence with previous 2-year followup report of Northen et al. [41]; but long-term side effects on mothers and infants should be considered in further investigations [42, 43].

Based on this randomized clinical trials finding, application of vaginal progesterone has advantages in both maternal and neonatal indexes, while the exact mechanism remains unclear.

## Figures and Tables

**Table 1:** Comparison of demographic data between two groups of patients.

Parameters	Progesterone group	No-drug group	*P* value
Mean age ± SD (years)	24.2 ± 3.7	25.4 ± 2.9	0.41
Mean initial gestational age ± SD (years)	32.2 ± 2.8	32.7 ± 2.6	0.89
Mean length of cervix ± SD (cm)	1.8 ± 0.3	1.7 ± 0.4	0.70
Mean dilatation of cervix ± SD (cm)	1.5 ± 0.2	1.6 ± 0.3	0.91

**Table 2:** Comparison of maternal outcomes between two groups of patients.

Parameters	Progesterone group (*N* = 72)	No-drug group (*N* = 72)	*P* value
Mean gestational age at delivery ± SD (year)	36.2 ± 1.4	34.1 ± 1.5	0.039
Mean time of postponing delivery ± SD (week)	4.0 ± 1.5	1.4 ± 0.2	0.048
Mean of cervical length and dilatation ± SD	1.8 ± 0.3	1.7 ± 0.4	0.71
Mean of cervical length and dilatation ± SD	1.5 ± 0.2	1.6 ± 0.3	0.92
Type of delivery (Cesarean/NVD)	16/56	19/53	0.058

**Table 3:** Comparison of neonatal outcomes between two groups of patients.

Parameters	Progesterone group (*N* = 72)	No-drug group (*N* = 72)	*P* value
Mean weight of neonate ± SD (Kg)	2950.6 ± 420.3	2628.0 ± 385.1	<0.001
Number of low birth weight (%)	8 (11.1)	26 (36.1)	<0.001
Number of neonates admitted in NICU (%)	6 (8.3)	17 (23.6)	<0.001
Number of neonatal deaths (%)	3 (4.2)	8 (11.1)	0.08
